# The Effects of In Vivo Exposure to Copper Oxide Nanoparticles on the Gut Microbiome, Host Immunity, and Susceptibility to a Bacterial Infection in Earthworms

**DOI:** 10.3390/nano10071337

**Published:** 2020-07-09

**Authors:** Elmer Swart, Jiri Dvorak, Szabolcs Hernádi, Tim Goodall, Peter Kille, David Spurgeon, Claus Svendsen, Petra Prochazkova

**Affiliations:** 1 UK Centre for Ecology and Hydrology, Maclean Building, Benson Lane, Wallingford OX10 8BB, UK; timgoo@ceh.ac.uk (T.G.); dasp@ceh.ac.uk (D.S.); 2 Laboratory of Cellular and Molecular Immunology, Institute of Microbiology of the Czech Academy of Sciences, Videnska 1083, 142 20 Prague 4, Czech Republic; dvorak@biomed.cas.cz (J.D.); kohler@biomed.cas.cz (P.P.); 3 School of Biosciences, Cardiff University, Sir Martin Evans Building, Museum Avenue, Cardiff CF10 3AX, UK; hernadis1@cardiff.ac.uk (S.H.); kille@cardiff.ac.uk (P.K.)

**Keywords:** innate immunity, infection, microbiome, survival, nanomaterials, nanoparticles, copper, earthworms, *Eisenia fetida*

## Abstract

Nanomaterials (NMs) can interact with the innate immunity of organisms. It remains, however, unclear whether these interactions can compromise the immune functioning of the host when faced with a disease threat. Co-exposure with pathogens is thus a powerful approach to assess the immuno-safety of NMs. In this paper, we studied the impacts of in vivo exposure to a biocidal NM on the gut microbiome, host immune responses, and susceptibility of the host to a bacterial challenge in an earthworm. *Eisenia fetida* were exposed to CuO-nanoparticles in soil for 28 days, after which the earthworms were challenged with the soil bacterium *Bacillus subtilis*. Immune responses were monitored by measuring mRNA levels of known earthworm immune genes. Effects of treatments on the gut microbiome were also assessed to link microbiome changes to immune responses. Treatments caused a shift in the earthworm gut microbiome. Despite these effects, no impacts of treatment on the expression of earthworm immune markers were recorded. The methodological approach applied in this paper provides a useful framework for improved assessment of immuno-safety of NMs. In addition, we highlight the need to investigate time as a factor in earthworm immune responses to NM exposure.

## 1. Introduction

Nanomaterials (NMs) are increasingly used in various applications including surface coatings, biocide pesticides, and electronics [1,2]. The potential risks of NM to human health and the environment have long been identified [3,4,5]. Over the last decade, research has provided vast amounts of toxicity data that have reduced many of the initial uncertainties around NM risk. There are, however, still some aspects that need further investigation. One of these remaining issues relates to the immuno-safety of NMs [6,7,8]. Owing to their particulate nature, NMs have an increased potential to interact with the innate immune system of organisms [9,10,11,12] and to induce both pro- and anti-inflammatory responses [13]. Most of the current research to investigate such effects has used in vitro models to characterize NM–immune interactions. Although these studies have provided crucial information on how immune systems may interact with NM, it remains unclear how responses in vitro will translate to in vivo effects. Further, immuno-modulation by NM does not necessarily indicate that the immune system is being compromised. In fact, immune reactions are part of a healthy response by the host towards foreign objects. In order to assess whether NMs actually compromise host immunity, co-exposure with infectious pathogens is necessary [7,8].

A major application of NMs is as antimicrobial agents in pesticides and coatings. The effects of biocidal NM on soil microbial communities have been relatively well studied [14,15,16]. There are, however, uncertainties concerning the impact of NMs on microbes associated with plants and animals (commonly referred to as ‘microbiome’) [17]. Common roles of the microbiome in host health include the provision of essential nutrients and aiding in digestion, while the role of the microbiome in host immunity is now increasingly being recognized [18,19,20]. Microbes associated with mucosal surfaces can contribute to immunity by providing resistance against invading pathogens [21,22,23] and by stimulating the release of antimicrobial peptides by the host [24]. Disruption of the healthy microbiome by, for example, chemical exposure, can lead to reduced immune functioning and reduced survival of bacterial infections [25,26,27]. Previous studies have shown that, when NMs alter the microbiome of animals, the expression of host immune genes can also change [28,29,30,31]. Host immunity and the microbiome are thus a complex and integrally linked system, and it is thus important to include microbiome analysis in the immuno-safety assessment of NM. Invertebrate animals such as earthworms, bivalves, and sea urchins provide suitable models to study in vivo effects of NM on immune functioning under more realistic environmental conditions [31,32,33].

Earthworms provide crucial ecosystem services in soils through mixing, organic matter degradation of plant material, and nutrient cycling, and thereby contribute to enhanced crop production [34]. Living in soil, earthworms inhabit an environment with high microbial activity. To provide protection from pathogens, cellular immunity in earthworms is provided by immune cells called coelomocytes, which circulate the coelomic cavity. A crucial element of the innate immune system is the recognition of microorganism associated molecular patterns (MAMPs) by host pathogen recognition receptors (PRRs). A well described PRR in earthworms is coelomic cytolytic factor (CCF). This PRR, upon binding to specific MAMPs, induces the prophenoloxidase pathway, which ultimately leads to the production of antimicrobial factors [35,36,37]. Earthworm pathogens are also controlled by various humoral factors. One of these is lysozyme, an enzyme that can hydrolyse components of the cell wall of Gram-positive bacteria [38]. In the earthworms *Eisenia fetida* and *Eisenia andrei,* immunity is also supported by the humoral factors lysenin [39] and fetidin [40,41], the modes of antibacterial action of which are not fully understood [40,42]. Recent work shows that changes in gene expression of these immune factors can be used as a marker of immune-modulation in earthworms [38,43,44]. In vitro studies have shown that exposure to NM can also alter the expression of earthworm immune genes [45,46]. Effects of in vivo exposure to NM earthworm immune system regulation have not been studied extensively [33] and it remains uncertain whether NM exposure can compromise earthworm immunity by affecting the host susceptibility to infections.

The earthworm gut microbiome has been relatively well described. Gut communities have been identified as being composed of both transient bacteria associated with ingested soil and food [47] and resident bacteria more closely associated to intestinal surfaces [48,49,50]. Loss of some core earthworm symbionts can lead to reduced host fitness and juvenile development [51,52]. Environmental pollutants can alter the microbiome of earthworms [53,54,55,56] and lead to the loss of core symbionts important to host health [57]. An increasing body of literature now indicates that exposure to NMs can also disrupt the microbiome of soil invertebrates [58,59,60]. In the earthworm *Enchytraeus crypticus*, for example, exposure to CuO-NP can significantly reduce the abundance of core intestinal *Plantomycetes* bacteria [58]. The interplay between microbiome, NMs, and host immunity in earthworms, however, has not been studied. Disruption of host–microbiome interactions can be expected for chemicals that are designed to target microbes (biocides). In agriculture, copper-based NM formulations are being developed for biocidal applications [61]. In widespread application, there is thus the potential for such NMs to negatively affect earthworms through alterations to their microbiome structure.

Because of the integrate link between the microbiome, host immunity, and health status of animals, studies on the immuno-safety assessment of NM require a holistic approach. This paper aims to study whether exposure to copper oxide nanoparticles (NPs) has an effect on the gut microbiome structure, host immunity, and susceptibility to a bacterial infection in earthworms. For this purpose, earthworms were exposed in soil to concentrations of copper forms known to alter the earthworm gut microbiome for a duration of 28 days. The earthworms were subsequently removed from soils and challenged with the bacterium *Bacillus subtilis* for a further four days. The effects of the bacterial challenge were assessed by looking at survival and tissue damage, and by measuring mRNA levels of known immune markers. An analysis of the gut microbiome was concurrently conducted through a metabarcoding approach to link the effects on microbiomes to immune responses. The effects of NP were compared to those of metal salts, to test whether any effects were attributed to particles or ions. We hypothesized that (i) earthworms that have their microbiome changed through exposure to CuO-NP and copper salts are more susceptible to a bacterial infection; and (ii) exposure to CuO-NP, copper salts, and the proceeding bacterial challenge will have an effect on the gene expression of tested immune markers, in line with previous studies [44,62]. The holistic methodological approach applied in this paper provides a useful framework for improved assessment of immuno-safety of NMs.

## 2. Materials and Methods

### 2.1. Test Organism, Test Chemicals, and Soil Spiking

*Eisenia fetida* were reared at 20 °C in a medium consisting of loamy top soil, composted bark, and garden compost in 1:1:1 ratio by volume basis. Earthworms were fed with field collected horse manure from horses grazing on unpolluted pastures and free from recent medical treatment. All earthworms used in the experiment had a stripe patterned outer body characteristic of *E. fetida* with fully developed clitella and were within a weight range between 300 and 600 mg.

Molecular grade CuCl_2_·2H_2_O was supplied by Sigma-Aldrich (Poole, UK). CuO-NPs were manufactured by Promethean Particle Ltd. (Nottingham, UK) and were dispersed in water. Nanoparticles were cuboid in shape, with a stated mean dimension of 20 by 50 nm. Size distributions of NPs were determined with nanoparticle tracking analysis using a Nanosight (Malvern Instruments, Salisbury, UK). Derived mean and modal dimensions were 183 nm (±SE 5.2). Zeta potential of CuO-NPs (33 mV ± SD 0.3 mV) was determined using phase analysis light scattering using a Malvern Zetasize Nano ZS.

All exposures in soil were conducted in LUFA 2.2 natural soil (LUFA-Spreyer, Germany), a sandy loam soil widely used in ecotoxicological testing. Test soils were spiked with a nominal concentration of CuO-NP (160 mg·kg^−1^ d.w. soil) or CuCl_2_ (160 mg·kg^−1^ d.w. soil) or a negative control (0 mg·kg^−1^ d.w. soil). Test concentrations were based on a previous study that showed changes in the microbiome structure and loss of core symbionts at these copper concentrations [56]. CuO-NP treated soils were spiked one day before the initiation of the exposure. CuCl_2_ treated soils were spiked five days before the start of the exposure to allow the metal speciation in the soil to reach a quasi-equilibrium [63]. A control consisting of the liquid carrier of the CuO-NP dispersion was not included, as previous work has established that the liquid carrier of this NP dispersion is not toxic to earthworms and does not alter the earthworm microbiome structure [56].

Mixing of the chemicals with the soil was done for each treatment following Waalewijn-Kool et al. [64]. Briefly, the total amounts of CuO-NP and CuCl_2_ required for all replicates were dissolved in 60 mL of de-ionised water. These stock solutions were then each mixed with 250 g of d.w. LUFA 2.2 soil using a spatula. The mixture was then thoroughly mixed with the remaining soil and subsequently wetted with de-ionised water to reach 55% of the water holding capacity (WHC) before final mixing. The soil mixtures were divided into replicates each consisting out of a 60 g w.w. aliquot in a 100 mL clear plastic round tub.

### 2.2. Overview of the Experimental Design

**Figure 1:**
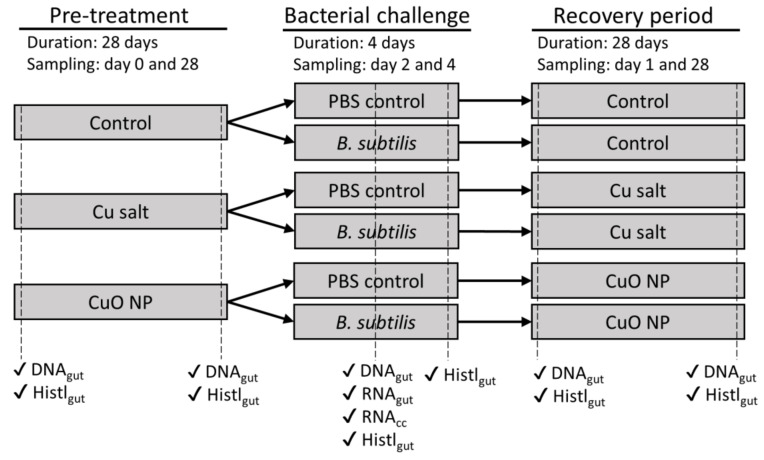
Schematic overview of the study design and collected samples. Dashed lines indicates sampling points. Tick marks indicate the samples collected at the respective sampling point. ‘DNA_gut_’ indicates sampling of DNA from gut tissue for microbiome analysis; ‘RNA_gut_’ and ‘RNA_cc_’ indicate sampling of RNA for gene expression analysis from gut tissue and coelomic fluid, respectively; and ‘Histl_gut_’ indicates sampling of gut tissue for histological analysis. NP, nanoparticle; PBS, phosphate buffered saline.

Earthworm were initially exposed to a pre-treatment of either CuO-NP, CuCl_2_, or a negative control for 28 days, after which time they were removed from soil and challenged with either *Bacillus subtilis* or a negative control for four days (Figure 1). *B. subtilis* was chosen as a model pathogen for the bacterial challenge on the basis of a pilot experiment, which showed that the coelomic fluid of *E. fetida* had inhibiting effects on the growth of this bacterium, possibly indicating an effective immune response by cellular or humoral components (Figure S1). After this bacterial challenge, earthworms were returned to their original soil for recovery for another 28 days, during which time they were periodically sampled for analysis. Details of each of the three experimental steps are described below.

### 2.3. Pre-Treatment Exposure

Prior to the start of the pre-treatment exposure, earthworms were acclimatized to LUFA 2.2 soil for two weeks under the same conditions as the main exposure assay. Before exposure initiation, earthworms were rinsed and weighed. To start the test, one adult *E. fetida* was added to each test replicate. The pre-treatment exposure was conducted at 20 °C for 28 days. Once a week, each replicate received 0.5 g of spiked horse manure on a d.w. basis as food. At the end of the exposure, earthworms from all replicates were removed from soil, rinsed, and weighed. Collected earthworms were subsequently depurated on wetted filter paper to allow egestion of their gut content for two days before bacterial challenge.

### 2.4. Bacterial Challenge

Following depuration, each earthworm was challenged in a petri dish with either *Bacillus subtilis* (*B. subtilis* subsp. *subtilis*, CCM2217; Czech Collection of Microorganisms, Brno, Czech Republic) or a negative control in a medium consisting of re-wetted paper pellets. The pellets were re-wetted using either 10 mL of phosphate buffered saline (PBS) containing 5 × 10^8^ *B. subtilis* cells per mL or 10 mL PBS only. *B. subtilis* cultures used for the challenge were in exponential growth phase at the time of the start of the challenge. Cell concentration was determined by measuring OD_600_. The bacterial challenge was initiated by placing a depurated earthworm into the prepared Petri dish. The bacterial challenge was conducted at 20 °C in dark conditions for four days. Earthworm survival was monitored at day one, two, and four.

### 2.5. Recovery Period

After the bacterial challenge, earthworms were rinsed, weighed, and subsequently returned to the original soil exposure replicates to assess responses to exposure after the bacterial challenge. The recovery exposure was conducted at 20 °C and lasted 28 days. Earthworms were fed with 0.5 g of d.w. spiked horse manure every week.

### 2.6. Sample Points

Six sampling points were used: ‘pre-treatment day 0’, ‘pre-treatment day 28’, ‘bacterial challenge day 2’, ‘bacterial challenge day 4’, ‘recovery period day 1’, and ‘recovery period day 28’ (Figure 1). At every sampling point, except ‘bacterial challenge day 4’, gut tissue and coelomic fluid from five earthworms were collected (see below). At every sampling point, one additional earthworm was collected for histological analysis (see below). All earthworms collected from soil exposure replicates (i.e., ‘pre-treatment day 0’, ‘pre-treatment day 28’, ‘recovery period day 1’, and ‘recovery period day 28’) were depurated for two days prior to sampling. Earthworms collected at day two of the bacterial challenge were rinsed in de-ionised water, but not depurated and immediately dissected. At the end of the pre-treatment exposure, 10 g w.w. soil was collected for metal analysis.

### 2.7. Sampling of Gut Tissue and Coelomic Fluid

Each depurated earthworm was placed in a petri dish containing 500 µL PBS and coelomic fluid was extruded by electrification for 5 s using a 4.5 V battery. The mixture of coelomic fluid and PBS was collected and mixed with 500 µL of 2× RNA/DNA Shield (Zymo Research, Irvine, CA, USA) and placed in a lysis tube. The extruded earthworm was subsequently euthanized in pure ethanol and the midgut (spanning 20 segments posterior to the clitellum) was dissected using sterile equipment. Small incisions were made along the length of the midgut and rinsed in 1 mL PBS for 1 min using a vortex to facilitate removal of any residual soil, and subsequently placed in a lysis tube. All lysis tubes were bead beaten using an MP FastPrep-24^TM^ set at 4.5 m/s for one minute. Lysis tubes were placed at 6 °C overnight and subsequently stored at −20 °C until DNA or RNA extraction.

### 2.8. Histological Analysis

Earthworms collected for histological analysis were fixed and processed according to Dvorak et al. [44]. Briefly, a whole body sample spanning a 10 segment region posterior to the clitellum was fixed in 4% paraformaldehyde overnight, dehydrated, and embedded in paraffin. For each sample, three 2 µm sections were cut using a microtome, dried overnight, deparaffinised using xylene, rehydrated, and stained using hematoxylin/eosin following Kiernan [65]. Sections were visually inspected for tissue integrity using a light microscope. Damage to the gut epithelium and chloragogen tissue was scored using an ordinal scoring method with four categories ((1) no effects, (2) mild effects, (3) moderate effects, and (4) severe effects), following Gibson-Corley et al. [66].

### 2.9. Soil Metal Measurements

Soil copper concentrations were measured in 130 mg of d.w. soil, which was mixed with a 4:1 mixture of nitric acid and hydrochloric acid on a volume basis and digested for seven hours at 150 °C. Copper concentration was determined using atomic absorbance spectrometry at the Vrije Universiteit Amsterdam (The Netherlands). Copper recovery from exposure soils was on average 60.4% and 71.3% for CuO-NP and CuCl_2_ spiked soils, respectively. The lack of full recovery may be linked to the loss of copper during preparation of stocks solutions and owing to heterogeneity in the distribution of copper forms in soils.

### 2.10. DNA and RNA Extraction and cDNA Synthesis Procedure

DNA was extracted from gut tissue and soil using a Quick-DNA Fecal/Soil Microbe Miniprep Kit (Zymo Research) according to the protocol supplied by the manufacturer. RNA was extracted from gut tissue and coelomic fluid using Quick-RNA™ Miniprep Kit (Zymo Research) following the protocol supplied by the manufacturer and included a DNA removal step using DNase. Visual inspection through agarose gel electrophoresis under denaturing conditions verified that the RNA in all samples was not degraded. Extracted RNA was subjected to a further clean-up using a Clean and Concentrator^TM^-5 kit (Zymo Research). RNA quantity of the cleaned samples was determined using Qubit™ RNA HS Assay Kit (ThermoFisher Scientific, Waltham, MA, USA). Per sample, 250 ng of RNA was reverse transcribed to cDNA using Reverse Transcription System A3500 (Promega, Madison, WI, USA) following the standard protocol supplied by the manufacturer.

### 2.11. Earthworm Genotyping

Gene expression analysis relies on accurate binding of primers to target genomic regions. Genetic variation in binding sites between different individuals is likely to reduce the efficacy to elucidate patterns of gene expression. Within species genetic diversity in earthworms is high [67,68,69]. On the basis of cytochrome c oxidase I (COI) sequence similarities, two distinct genetic *E. fetida* clades have so far been recognised [70]. To screen whether earthworms used in this experiment were part of a single genetic clade, earthworms sampled at day two of the bacterial challenge were genotyped by amplification and sequencing of the mitochondrial COI DNA. PCR reactions were set up using forward primer COI_1490 (5′-GGTCAACAAATCATAAAGATATTGG-3′) and reverse primer HCO_2189 (5′-TAAACTTCAGGGTGACCAAAAAATCA-3′) [71] using One*Taq*^®^ Hot Start polymerase and reaction buffer (New England Biolabs) using the following programme: initial denaturation at 94 °C for 2 min followed by 35 cycles of (1) denaturing at 94 °C for 30 s, (2) annealing at 47 °C for 30 s, and (3) extension at 68 °C for 1 min, followed by a final extension step at 68 °C for 10 min. Amplification of a single fragment was verified through gel electrophoresis. PCR products were cleaned using a QIAquick PCR purification kit (QIAGEN) and DNA quantity was assessed using a Qubit dsDNA HS Assay Kit (ThemoFisher Scientific). Then, 7.5 ng of PCR product was sequenced using Sanger sequencing using 3.2 pg of the forward primer at the University of Birmingham (UK). Sanger sequences were submitted to the National Center for Biotechnology Information (NCBI) BLASTn for taxonomical assignment. Pairwise alignment of sequences was performed using MUSCLE alignment in Geneious 9.1.8. Ambiguous bases and erroneous inserts were manually resolved and low quality ends of sequences were trimmed. The remaining 632 bp alignment was used as input for genetic analysis in MEGA software v7. Gamma-distributed Hasegawa, Kishino, and Yano model was calculated to best fit the data and used to calculate a maximum-likelihood phylogenetic tree using 500 bootstraps. Pairwise between groups genetic distance was calculated in MEGA.

Phylogenetic analysis on the COI gene revealed the existence of three separate genetic clusters. From each cluster, five samples were selected and subjected to further genetic analysis through random amplification of polymorphic DNA (RAPD). The RAPD reactions were conducted using the primer 5′-CAGGCCCTTC-3′ [72] and One*Taq*^®^ polymerase and reaction buffer (New England Biolabs) following the thermal cycling programme: initial denaturation at 94 °C for 2 min followed by 35 cycles of (1) denaturing at 94 °C for 1 min, (2) annealing at 37 °C for 1 min, and (3) extension at 68 °C for 2 min, followed by a final extension step at 68 °C for 10 min. Genomic DNA extracted from the earthworm *Lumbricus rubellus* was used as outgroup. PCR product was run on a 1.5% agarose gel for three hours at 120 V using 1 kb HyperLadder (Bioline, London, UK) as reference. Band patterns were manually scored in a blind manner. Rooted neighbourhood-joining tree was calculated in R 3.5.0 (www.r-project.org) using the package “ape” [73].

### 2.12. Gut and Soil 16S Sequencing Metagenomics Bioinformatics

The prokaryotic community in genomic DNA extracted from soil and gut tissue was determined by PCR amplification and sequencing following the method outlined by Kozich et al. [74]. Briefly, a ~555 bp fragment spanning the V3–V4 region of the 16S-rRNA gene was amplified using the forward primer 5′-CCTACGGGAGGCAGCAG-3′ and reverse primer 5′-GGACTACHVGGGTWTCTAAT-3′, each modified with the addition of a sequencing primer, an indexing region, and an Illumina flow-cell adaptor such that each sample was uniquely barcoded. PCR amplification was done using Q5^®^ High-Fidelity DNA Polymerase and reaction buffer (New England Biolabs, Ipswich, MA, USA) using the following programme: initial denaturing at 95 °C for 2 min, followed by 30 cycles of (1) denaturing at 95 °C for 30 s, (2) annealing at 55 °C for 15 s, and (3) extension at 72 °C for 40 s, followed by a final extension step at 72 °C for 10 min. Gel electrophoresis was used to verify amplification of a single product. PCR product was normalized using SequalPrep™ Normalization Plate Kit (ThemoFisher Scientific) and samples from each normalization plate were pooled. The pooled samples purified using QIAquick Gel Extraction Kit (QIAGEN, Venlo, The Netherlands). Gel extracted libraries were quantified using Qubit dsDNA HS Assay Kit (ThemoFisher Scientifc) and equimolary pooled and diluted to 7 pM. The pooled library was sequenced with 10% PhiX on a MiSeq using MiSeq Reagent Kit v3—600 cycles (Illumina, Inc., San Diego, CA, USA). The Illumina demultiplexed sequences were processes using the DADA2 bioinformatics pipeline [75] to generate an amplicon sequence table from the forward reads. DADA2 settings were maxEE(2), maxN(0), and truncQ(2). Sequences were trimmed to 290 bases. Sequences were dereplicated and the DADA2 core sequence variant inference algorithm was applied. Chimeric sequences were removed using removeBimeraDenovo default settings. Amplicon sequence variants (ASVs) were subjected to taxonomic assignment using assignTaxonomy at default settings and the Silva database [76]. ASVs assigned to mitochondria, chloroplasts, Archaea, Eukaryotes, and ASVs with unknown kingdom or phylum were removed from the dataset. Nucleotide sequence data have been submitted to NCBI and are available under submission number SUB7500125 as part of BioProject number PRJNA610159.

### 2.13. Quantitative PCR

Quantitative PCR was used to determine differential levels of mRNA of several earthworm immune genes in gut tissue and coelomic fluid (Table 1). Both tissues were screened for coelomic cytolytic factor (CCF), lysozyme, and lysenin/fetidin. Primer pairs were mapped against reference transcriptomes of both *E. fetida* and *E. andrei* to estimate the binding potential to all known allelic variants. Primer pairs targeting CCF were designed to both *E. fetida* and the *E. andrei* versions of the CCF gene using Primer 3. Amplification efficiency of primer pairs was verified through serial dilution and was between 90% and 110% for all pairs. Amplification of the target fragment was verified by Sanger sequencing of the PCR products. qPCR reactions were conducted using GoTaq^®^ qPCR Master Mix (Promega) in a 20 µL reaction volume using 6.25 ng of cDNA as input. qPCR was performed using a Roche LightCycler^®^ 480II with PCR conditions: initial denaturation at 95 °C for 3 min, followed by 40 cycles of (1) denaturation at 95 °C for 10 s and (2) annealing and extension at 60 °C for 30 s. Melt curve analysis was conducted to verify single PCR product. Changes in gene expression were calculated using the 2^−ΔΔCt^ method [77]. EF1α was used as a reference gene for the normalization of the target immune genes. Log2 fold change was expressed in relation to the negative control (earthworms exposed to control soils in the pre-exposure and PBS in the bacterial challenge).

### 2.14. Statistical Analysis

All data analysis was done in R (www.r-project.org). Non-metric dimensional scaling (NMDS), distance-based redundancy analysis (db-rda) (using Bray–Curtis distance matrix), permutational analysis of variance (Permanova), and calculation of diversity indices were conducted using the R package ‘vegan’ [78] using datasets rarefied to 4959 reads per sample with removal of samples below this threshold. Differences between treatments in diversity indices and gene expression values were tested using two-way analysis of variance (2w-ANOVA) and Tukey’s post hoc test. Differential abundance analysis of bacterial taxa was done using Kruskal–Wallis Rank Sum Test and Mann–Whitney test using datasets rarefied to 2448 reads. For the differential abundance analysis, rarefication to this lower read number was done to prevent losing replicates and, therefore, statistical power.

## 3. Results

### 3.1. Earthworm Population Genotypes

**Figure 2:**
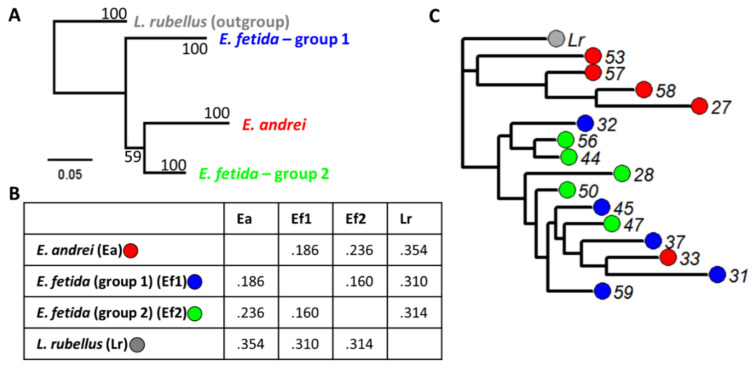
Phylogenetic trees showing relationship between cytochrome c oxidase I (COI) cluster and genetic distances between COI clusters. (**A**) Maximum-likelihood phylogenetic tree of showing phylogenetic relation between the three COI clusters. Samples are collapsed to COI cluster level, see Figure S2 for full tree. Bootstrap values are derived using 500 bootstraps. (**B**) Table with genetic distances between the three COI clusters. (**C**) Rooted neighbourhood-joining tree based on random amplification of polymorphic DNA (RAPD) profiles with *L. rubellus* (*Lr*) as outgroup. Different number indicate sample number. Colours indicate COI grouping.

Sequencing of the COI gene from earthworms sampled during the bacterial challenge indicated three separate genetic clusters (Figure 2A). Local alignment using NCBI BLASTn indicated that one of those clusters was most similar to *E. andrei* COI, while the COI of the two other clusters aligned best with *E. fetida* COI. The two *E. fetida* COI sub-clusters did not group together in the phylogenetic tree. However, the overall genetic distance between the two *E. fetida* COI clusters was smaller (0.160) than that between the *E. andrei* COI cluster and *E. fetida* COI cluster 1 (0.186) (Figure 2B). RAPD analysis based on 26 polymorphic markers indicated the existence of two genetic clusters (Figure 2C). One of these clusters consisted of individuals carrying an *E. andrei* COI gene copy. The other cluster comprised individuals carrying an *E. fetida* COI copy and one COI assigned *E. andrei* individual.

### 3.2. The Effects of Pre-Treatment Exposure and Bacterial Challenge on the Gut Microbiome

The earthworm gut community at the start of the pre-treatment was composed of a consortium of bacteria comparable to that found in previous studies [56,79]. The gut community was dominated by *Verminephrobacter* (*Proteobacteria*), ‘*Candidatus* Lumbricincola’ (*Mollicutes*), a member of the *Spirochaetaceae* family (*Spirochaetes*), and multiple ASVs belonging to the genus *Aeromonas* (*Proteobacteria*) (Figure S3). Transfer of earthworms from culture soil (‘pre-treatment day 0’) to LUFA control soils (‘pre-treatment day 28’) did not significantly alter the total community structure in the gut (Permanova: *F*(1,8) = 1.059, *p* = 0.356) (Figure S3) nor Shannon diversity (*F*(1,8) = 0.882, *p* = 0.375) or species richness (*F*(1,8) = 0.074, *p* = 0.375) (Table S1). Average Shannon diversity and richness across all replicates were 3.0 (±SD 1.1) and 239 (±SD 146), respectively.

**Figure 3:**
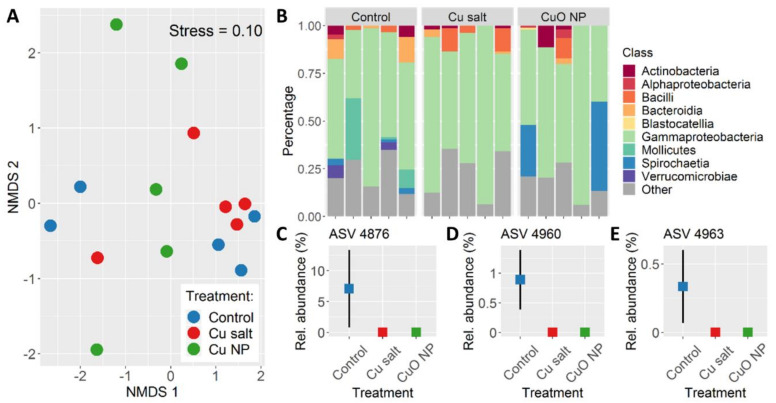
Bacterial community composition and structure of earthworm gut samples at the end of the pre-treatment exposure (i.e., ‘pre-treatment day 28’). (**A**) Plot of non-metric dimensional scaling (NMDS) showing ordination of samples at amplicon sequence variant (ASV) level. (**B**) Relative abundance of dominant ASV at class level per sample. All ASVs with a relative abundance <1% of the total community are grouped under “Other”. Mean relative abundance (±se) per treatment as percentage of total community of (**C**) ‘*Candidatus* Lumbricincola’ (ASV 4876), (**D**) *Luteolibacter pohnpeiensis* (ASV 4960), and (**E**) *Luetolibacter* (ASV 4963).

At the end of the pre-treatment exposure (i.e., ‘pre-treatment day 28’), there were no significant differences in overall community structure between the treatment groups (Permanova: *F*(2,12) = 1.252, *p* = 0.256) (Figure 3A). Bacterial diversity was also not affected by pre-treatment exposure (Shannon: (*F*(2,12) = 0.176, *p* = 0.84; richness: *F*(2,12) = 1.695, *p* = 0.225) and was on average 2.5 (±0.9) (Shannon) and 189 (±114) (richness) (Table S1). Among the most abundant gut bacteria (ASVs with a relative abundance >1% in any of the samples), three ASVs were significantly negatively affected by exposure to both copper forms (Kruskal–Wallis: *p* < 0.05). These ASVs included ‘*Candidatus* Lumbricinola’ (ASV 4876) and two ASVs belonging to *Luteolibacter* (ASV 4960 and 4963) (Figure 3C–E). These taxa together comprised an average of 8% of the total community in controls, but were at or below the limit of detection in the copper-treated earthworms.

In earthworms that were sampled at ‘bacterial challenge day 2’, treatment (i.e., pre-treatment exposure followed and bacterial challenge combined) had a nearly statistically significant effect on the bacterial community composition in earthworms, with treatment explaining 24% of the total variance (Permanova: *F*(5,23) = 1.423, *p* = 0.075) (Figure S4). Pre-treatment exposure and bacterial challenge treatment alone explained 10% (Permanova: *F*(2,26) = 1.453, *p* = 0.108) and 4% of the total variance (Permanova: *F*(2,26) = 1.222, *p* = 0.265), respectively (Table 2). No significant effect of treatment on diversity indices, which averaged 1.95 (±0.6) (Shannon) and 57 (±22) (richness) across all samples, was found (Table S1). Among the most dominant gut bacteria (ASVs with relative abundance >1%), ‘*Candidatus* Lumbricincola’ (ASV 4876) relative abundance was significantly negatively affected by copper treatment (Figure S4D) (Kruskal–Wallis: *X*^2^(5) = 12.1, *p* < 0.05), while *Aeromonas* (ASV 10149) was positively affected (Figure S4D) (Kruskal–Wallis: *X*^2^(5) = 14.0, *p* < 0.05). 

### 3.3. Relation between Earthworm Genotype and Bacterial Community Structure

**Figure 4:**
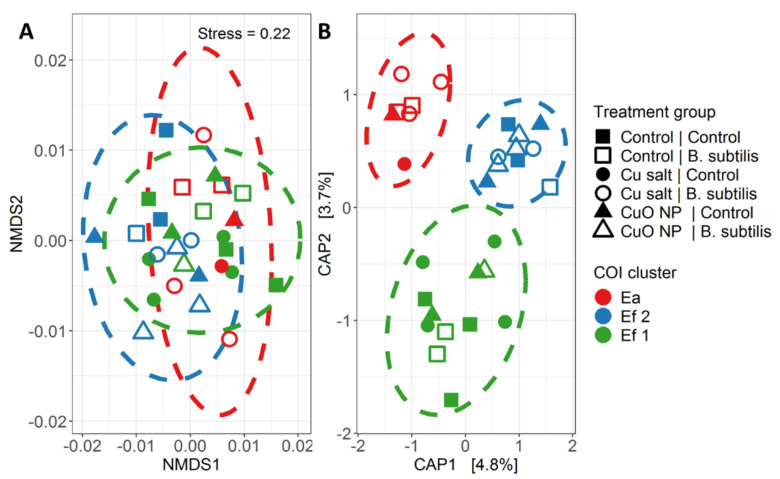
(**A**) NMDS plot showing ordination of ‘bacterial challenge day 2’ samples. (**B**) First two-axis of distance based redundancy analysis (db-rda) including ‘pre-treatment exposure’, ‘bacterial challenge treatment’, and ‘genotype’ as explanatory variables. Percentage following axis labels in (**B**) indicate percentage of total inertia explained by the respective axis. In both figures, different colours indicate different COI genotypes, while different shapes and filling indicate different treatments. Ellipses indicate 90% confidence interval (CI) of the respective COI cluster. In the legend, the text before the vertical bar indicates ‘pre-treatment exposure’ and the text following the vertical bar indicates ‘bacterial challenge treatment’. ‘Ea’ (*E. andrei*), ‘Ef1’ (*E. fetida* 1), and ‘Ef2’ (*E. fetida* 2) indicate COI genotype.

Permanova and db-rda indicated that genotype was a better predictor for the bacterial community composition than either pre-treatment exposure or bacterial challenge treatment (Table 2) with samples clustering primarily by COI genotype (Figure 4). After removal of the variation associated to COI genotype using partial db-rda models, the effect of pre-treatment and bacterial challenge treatment on community composition was still not significant (Table 2).

### 3.4. Impact of Treatments on Bacillus Subtilis Abundance in Gut Tissue, Earthworm Survival, Tissue Integrity, and Immune Responses

**Figure 5:**
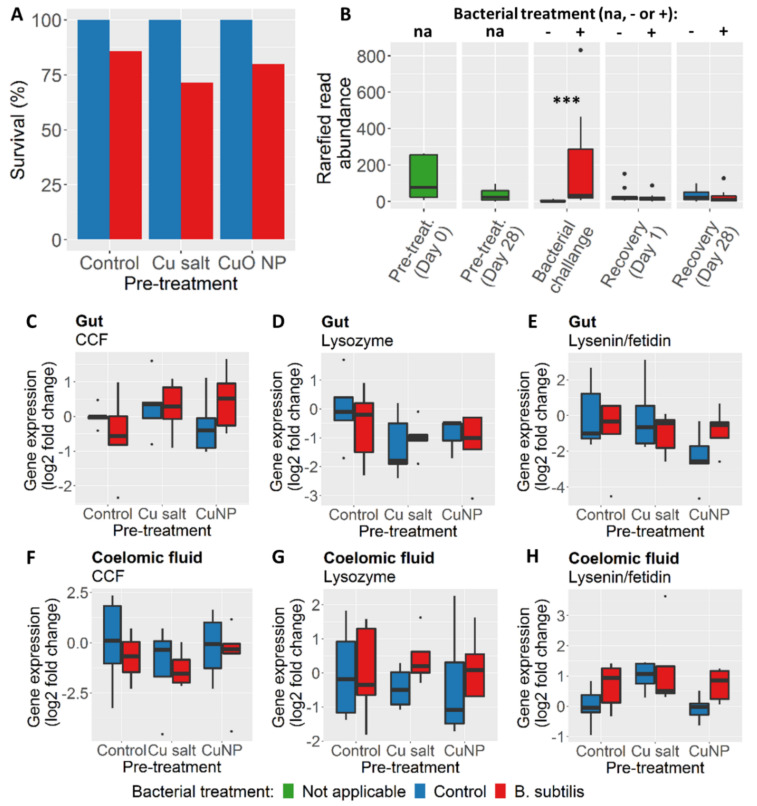
(**A**) Survival at day four of the bacterial challenge. (**B**) Boxplots of rarefied read abundance of *Bacillus* per sample point. Annotation at top of the graph indicates bacterial treatment: na (not applicable), − (PBS control), and + (*B. subtilis* treatment). Triple asterisks indicate statistical significance between bacterial challenge control and bacterial challenge treatment with *p* < 0.001. Relative high abundance of *Bacillus* at ‘pre-treatment day 0’ is driven by the two samples in that sample group (Figure S2). Boxplots of fold change in gene expression of earthworm immune responses in (**C–E**) gut tissue and (**F–H**) coelomic fluid at ‘bacterial challenge day 2’. All gene expression values are log2 fold change values derived through 2^−ΔΔCt^ method. Expression values represent fold changes of treatments compared to control animals (control pre-treatment + control bacterial challenge) and are normalized to the expression of the housekeeping gene EF1α. There were no significant differences in gene expression between groups for all tested genes (α = 0.05). Different colours in all panels indicate different bacterial challenge treatments. CCF, coelomic cytolytic factor.

All control earthworms exposed to the PBS control for four days survived. The survival of earthworms exposed to *B. subtilis* was on average 79% at day four, with no statistically significant effects of pre-treatment on the survival rate observed (*X*^2^(2) = 0.875, *p* = 0.646) (Figure 5A). Abundance of *Bacillus* in gut tissue from earthworms challenged with *B. subtilis* was significantly higher than in control animals, indicating successful inoculation (Figure 5B). No differences in the abundance of *Bacillus* in gut tissue were observed during the recovery period. Histological analysis indicated a possible effect of pre-treatment exposure with copper (in both forms) on the integrity of the gut epithelium and longitudinal muscle tissue. The average integrity scores in copper treatment were between 0.8 and 1.3 points higher than controls (Table S2). The effects of copper treatment were manifested as the thinning of the gut epithelium tissue lining as well as the thinning of muscle fibres (Figure S5). Gene expression levels were assessed through qPCR analysis, targeting several known earthworm immune genes using EF1α as reference gene (Figure S6). No significant effect of treatments was found on immune gene expression in both tissue types (2w-ANOVA: *p* > 0.05) (Figure 5C–H).

### 3.5. Relation between Earthworm Genotype and Gene Expression

No significant relation between gene expression and COI genotype was found, with the exception of lysozyme expression in coelomic fluid samples. For this gene, expression in the *E. andrei* COI genotype was marginally, but significantly higher than the *E. fetida* group 2 (2w-ANOVA: *F*(2,26) = 4.646, *p* < 0.05; Tukey’s post hoc test: *p* < 0.05), with the difference in mean fold change of 1.4 indicating a small magnitude effect (data not shown).

## 4. Discussion

Innate immunity provides a first line of defence against invading pathogens. NMs are known to interact with the immune system of organisms and can induce both pro- and anti-inflammatory responses [8,13]. NMs are developed and applied as antimicrobial agents in personal care products and in an agricultural setting. In many animals, microbial symbionts play an important role in host defence. Therefore, when animals are exposed to biocidal NM, disruption of their microbiome can be expected, which, accordingly, may lead to effects on host immunity. It remains unclear, however, whether NM exposure can compromise host immunity through effects on the microbiome when hosts are infected by pathogens. This paper aimed to study the impact of biocidal CuO-NPs and its ionic counterpart on the gut microbial community, host immune responses, and infection susceptibility in an earthworm.

Previous in vitro studies have shown that NPs can be taken up by earthworm immune cells (coelomocytes) [45,80,81], and can alter the expression of earthworm immune markers [45,46], leading to cellular toxicity [82,83]. In this study, in vivo exposure to metal biocides (in both metal salt and NP form) caused changes to microbiome structure, with several bacterial symbionts being negatively affected by exposure to copper. Following these exposures and microbiome changes, survival rates were unaffected when earthworms were challenged with a high dose of the soil bacterium *B. subtilis*. Histological analysis indicated possible tissue damage owing to copper exposure, although this analysis is based on observations for a limited number of samples, and thus further assessment of this response is needed. Overall, we found no evidence for altered infection susceptibility or altered immune gene regulation at biocide concentrations where the gut microbiome is already affected.

The lack of an immune response after NP exposure contrasts with the results of previous studies [45,46]. Hayashi and colleagues, for example, found that in vitro exposure in earthworm immune cells to Ag-NP significantly alters the temporal expression of immune genes [45]. Studies in other invertebrates, such as mussels and sea urchins, have also shown that both in vivo and in vitro exposure to NMs can modulate the immune system of these animals [30,31,84]. NMs that are released into the environment are likely to undergo transformations [85,86,87]. Uptake by organisms may further modify the shape, size, and form of NMs [88,89]. Therefore, the NMs that earthworm immune systems are exposed to in vivo may be different to the pristine forms that have often been used in in vitro studies. The discrepancy between the impact of NM on earthworm immune reactivity in vivo and those in vitro may thus be linked to the transformations of NMs in soil media and the resulting change in the immuno-reactivity of NMs.

Previous research has shown that the microbiome of animals can be altered by NM exposure. In rodents, for example, exposure to Ag-NP can negatively affect the abundance of *Firmicutes* and *Lactobacillus* and induce histological damage to intestinal tissue [28,29]. In soil invertebrates such as springtails and earthworms, metal NP exposure was also shown to alter intestinal microbiomes [58,59,60]. Exposure to Ag-NP in the springtail *Folsomia candida*, for example, negatively affects the abundance of *Firmicutes* and *Actinobacteria* in the gut of these soil invertebrates [59]. Although implications on host functioning were not further studied, other research shows that microbiome dysbiosis induced by environmental pollution can be associated to changes in the isotopic composition of springtails, suggesting an impact on nutrient turnover [90]. In this study, we found that the earthworm symbiont ‘*Candidatus* Lumbricincola’ is negatively affected by an exposure to copper forms. ‘*Candidatus* Lumbricincola’ is a bacterium exclusively associated to earthworms and has a possible role in the degradation of polysaccharides [47,91]. However, the implications of the near loss of this symbiont for the health and functioning of earthworms require further investigation.

Contrary to our hypothesis, a two-day exposure to a high bacterial level did not change the expression of known earthworm immune markers. Successful inoculation with the bacterium was confirmed by the mortality data and the abundance of *Bacillus* in gut tissue. The lack of an immune response at concentrations of bacteria at which 21% of the exposed individuals die is thus unexpected. Earthworms can show large variation in the expression of immune genes over time [45,92]. Timing of the expression of components of the immune system can be gene-specific [44], but can also depend on the specific pathogen to which the earthworm is exposed [92]. For example, in *E. andrei*, lysozyme is expressed within several hours of exposure to *Escherichia coli*, but only after 16 h following exposure to *B. subtilis* [38]. Similarly, in *E. andrei,* lysenin/fetidin has been reported to be upregulated after six hours in response to a *Staphylococcus aureus* exposure, but downregulated when exposed to *E. coli* [93]. The methodology adopted in this study was based on studies by Dvorak and colleagues, who showed that exposure to high levels of *B. subtilis* can induce changes in immune regulation of earthworms [43,44]. Discrepancy between measured immune responses in *E. fetida*, as reported in this study, and those measured in previous studies in a related species under similar condition show that earthworm immune responses are also species-dependent. Investigations into the molecular structure of the earthworm immune gene CCF in eight different species have shown that some earthworm species have a wider recognition capacity than others [37]. Even within closely related *Eisenia* spp., there are differences in the reactivity of immune genes and immunity related enzymatic activity [43]. These differences in immune reactivity between related earthworms may reflect differences in microbial environments, which may require niche-specific immune responses and lead to differing basal immune reactivities. Time as a factor in earthworm immune responses is thus not fully understood [33] and requires further species-specific investigation. Sufficient sampling over a time-course is needed to fully elucidate patterns of immune expression in earthworms under various environmental stressors and, in particular, to identify the specific points of highest upregulation for key genes.

Earthworm coelomocytes are composed of three subpopulations, each with a unique function [94,95] and molecular immune-expression profile [93,94,95,96]. This cellular complexity means that it is possible for different cell subpopulations to have different sensitivities to pollutant exposure [82,97]. Exposure to metals and xenobiotics, but also immunostimulants like LPS [98], has been demonstrated to change the ratio between the different coelomocyte cell subpopulations [82,97,99] and to alter the expression of earthworm immune markers [62]. In this study, coelomic fluid was extruded and sampled without separation of different cell subpopulations. Accordingly, the measured immune responses to *B. subtilis* exposure are an average of the expression levels of these genes across these different subpopulations. This, in combination with high variation between individuals in expression of some of the tested genes as previously reported [100], may limit the ability to elucidate differences in the patterns of gene expression within any individual cell subpopulation [82].

In this study, we found that 7 out of the 30 genotyped earthworms carried an *E. andrei* COI copy. Moreover, for the remaining *E. fetida* individuals, two COI clades were recorded. COI genotype, however, did not affect measured immune responses. The finding of clade structure for earthworms from the *E. fetida*/*E. andrei* complex is in agreement with previous studies [70,101,102,103]. *E. fetida* are phenotypically characterized by their stripped pigmentation pattern, whereas *E. andrei* are classically more uniformly red coloured. These two species were formally described as two different subspecies (e.g., *E. fetida fetida* and *E. fetida andrei*) [104], but, on the basis of crossbreeding experiments and differences in biochemical markers, were classified as separate species [105]. More recent research has shown that, in laboratory conditions, *E. fetida* and *E. andrei* can hybridize and produce fertile hybrid offspring [101,106]. Field studies also confirm that gene flow between these two species does occur [102]. Earthworms in this study were characterized by typical *E. fetida* pigmentation. Previous studies, however, report that pigmentation is not always a good predictor for COI genotype [101,103]. Here, RAPD profiling suggests that COI genotype does not always predict genomic variability, as indicated by the presence of an individual carrying an *E. andrei* COI copy within a clade consisting of *E. fetida* COI carrying individuals. The COI genotype was shown to be a better predictor for the bacterial community composition than any treatment. Host genetics is one of the components shaping the human gut microbiome [107,108], but similar relationships have also been observed in other animals such as mice [109] and invertebrates. In the water flea *Daphnia manga*, for example, host genotype shapes not only the structure, but also the functionality of the gut microbiome, in particular its ability to respond to toxic cyanobacteria [110]. The gut microbiome of *Eisenia* spp. is dominated by a consortium of bacteria that are vertically transmitted from parental animal to offspring [79,111]. The relation between COI genotype and gut microbiome structure may thus be linked to the concurrent maternal transmission of both mitochondria and bacterial symbionts.

## 5. Conclusions

We show that the microbiome of earthworms can change when exposed to a copper (in both NP and salt form). However, these biocide-mediated changes of the microbiome do not lead to altered susceptibility to a bacterial infection. Despite mortality when challenged with a bacterium, no effects of treatment on the measured earthworm immune markers were observed. The absence of an effect on immune function needs to be further validated by studies of gene expression using a greater time resolution of immune responses in earthworms and further identification of markers of immunity through, for example, full transcriptomic analysis. The methodological approach applied in this paper may guide future studies to improve the assessment of immuno-safety of NMs.

## Figures and Tables

**Table 1:** Details of primers targeting housekeeping gene (EF1α) and target immune system genes.

Primer Name	Target Gene(s)	F/R	Sequence (5′ → 3′)	Amplicon Length (bp)
EF1α_F	Elongation factor 1 alpha	F	ATCGGTCATGTCGATTCCGG	213
EF1α_R	Elongation factor 1 alpha	R	GGCAGTCTCGAACTTCCACA	
CCF_721F	Coelomic cytolytic factor	F	ACGACAACCGATACTGGCTG	193
CCF_914R	Coelomic cytolytic factor	R	CTCCCAGAAATCCACCCACC	
Lysfet_F	Lysenin/fetidin	F	TGGCCAGCTGCAACTCTT ^a^	177
Lysfet_R	Lysenin/fetidin	R	CCAGCGCTGTTTCGGATTAT ^a^	
Lysozyme_F	Lysozyme	F	GCCATTCCAAATCAAGGAAC ^a^	129
Lysozyme_R	Lysozyme	R	TAGGTACCGTAGCGCTTCAT ^a^	

^a^ from Dvorak et al. [43].

**Table 2:** Outcomes of models testing the relationship between bacterial community composition in the gut of earthworms sampled at ‘bacterial challenge day 2’ and different combinations of explanatory variables. Db-rda: distance based redundancy analysis using Bray–Curtis distance matrix and applying square root transformation and Wisconsin double standardization. Permanova: Permutational multivariate analysis of variance using Bray–Curtis distance matrix and 999 permutations. ‘Explained’ and ‘Unexplained’ represent in db-rda models the proportion of inertia either explained or unexplained by explanatory variables. In brackets (in the ‘Explained’ column), the fraction of the inertia that is conditioned is shown (i.e., removed). In Permanova models, ‘Explained’ and ‘Unexplained’ refer to the model R^2^ and residual R^2^ values.

Model Type	Model	Model Outcomes				
		Explanatory Variables	Explained	Un-Explained	*F*-Value	*p*-Value
Db-rda	Community~Pre-treatment + Bacterial treatment + Genotype	All	0.202	0.798	1.167	0.008 **
Db-rda	Community~Pre-treatment + Bacterial treatment	All	0.118	0.882	1.114	0.084
Db-rda	Community~Pre-treatment + Bacterial treatment + conditioned (Genotype)	Pre-treatment + Bacterial treatment	0.085 (0.117)	0.798	1.130	0.079
Db-rda	Community~Pre-treatment	Pre-treatment	0.041	0.959	1.159	0.103
Db-rda	Community~Bacterial treatment	Bacterial treatment	0.077	0.924	1.077	0.189
Db-rda	Community~Genotype	Genotype	0.085	0.915	1.205	0.021*
Permanova	Community~Pre-treatment + Bacterial treatment + Genotype	Pre-treatment	0.101	0.899	1.614	0.074
		Bacterial treatment	0.044	0.956	1.428	0.173
		Genotype	0.139	0.861	2.229	0.019 *
Permanova	Community~Pre-treatment + Bacterial treatment	Pre-treatment	0.101	0.899	1.470	0.128
		Bacterial treatment	0.044	0.956	1.300	0.220
Permanova	Community~Pre-treatment	Pre-treatment	0.101	0.899	1.453	0.108
Permanova	Community~Bacterial treatment	Bacterial treatment	0.043	0.957	1.221	0.265
Permanova	Community~Genotype	Genotype	0.167	0.833	2.598	0.004 **

* indicates *p* < 005, ** indicates *p* < 0.01.

## Supplementary Materials

The following are available online at https://www.mdpi.com/2079-4991/10/7/1337/s1, Figure S1: Growth over time of (**A**) *Bacillus subtilis*, (**B**) *Bacillus thuringiensis*, and (**C**) *Citrobacter rodentium* in LB medium (i.e., ‘Control’), filter sterilized culture medium consiting out of 800 µL LB medium and 200 µL 10x diluted coelomic fluid (‘Coelomic fluid’) or culture medium containing both diluted coelomic fluid and antibiotics (‘Antibiotics’); Figure S2: Maximum-likelihood phylogenetic tree of showing phylogenetic relation between all samples; Figure S3: Bacterial community composition and structure of earthworm gut samples from control replicates at start and end of pre-treatment; Figure S4: Bacterial community composition and structure of earthworm gut samples during bacterial challenge (i.e., ‘bacterial challenge day 2’); Figure S5: Examples of cross sections of *E. fetida* stained with hematoxylin/eosin representing a range of tissue integrity scores; Figure S6: Boxplots of C_p_ values of elongation factor 1 alpha (ef1α) per treatment in coelomic fluid and gut tissue; Table S1: Mean Shannon diversity index and bacterial richness (±SD) per sampling point and treatment group; Table S2: Integrity scores of gut epithelium and chloragogen tissue per sample point and treatment. Values are means of the scores of three sections from a single replicate.
