# Supplementary material for: The Effects of In Vivo Exposure to Copper Oxide Nanoparticles on the Gut Microbiome, Host Immunity, and Susceptibility to a Bacterial Infection in Earthworms
Source: Nanomaterials (Basel). 2020 Jul 9;10(7):1337. doi: 10.3390/nano10071337 (PMC7408611; doi:10.3390/nano10071337)
Supplement: Supplementary file 1 [file nanomaterials-10-01337-s001.pdf]

nanomaterials

Supplementary Materials

The Effects of In Vivo Exposure to Copper Oxide Nanoparticles on the Gut Microbiome, Host Immunity, and Susceptibility to a Bacterial Infection in Earthworms

Elmer Swart [1,*], Jiri Dvorak [2], Szabolcs Hernádi [3], Tim Goodall [1], Peter Kille [3], David Spurgeon [1], Claus Svendsen [1,*], and Petra Prochazkova [2]

[1] UK Centre for Ecology and Hydrology, Maclean Building, Benson Lane, Wallingford OX10 8BB, UK; timgoo@ceh.ac.uk (T.G.); dasp@ceh.ac.uk (D.S.)

[2] Laboratory of Cellular and Molecular Immunology, Institute of Microbiology of the Czech Academy of Sciences, 142 20 Videnska 1083, Prague 4, Czech Republic; dvorak@biomed.cas.cz (J.D.); kohler@biomed.cas.cz (P.P.)

[3] School of Biosciences, Cardiff University, Sir Martin Evans Building, Museum Avenue, Cardiff CF10 3AX, UK; hernadis1@cardiff.ac.uk (S.H.); kille@cardiff.ac.uk (P.K.)

* Correspondence: elmswa@ceh.ac.uk (E.S.); csv@ceh.ac.uk (C.S.); Tel: +44-(0)1491-692626 (E.S.); +44-(0)1491-692676 (C.S.)

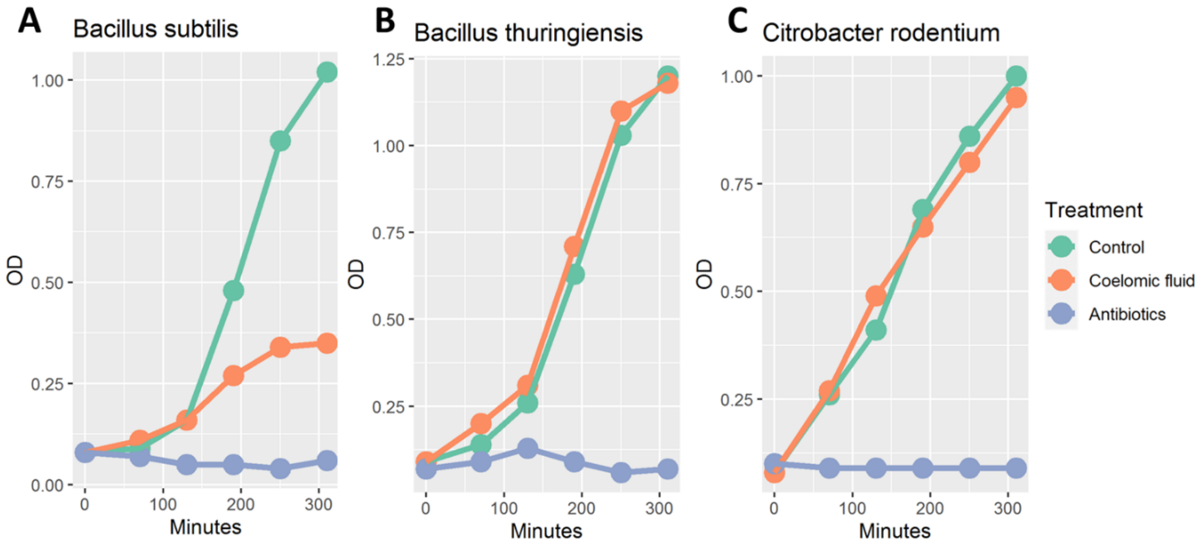

Figure S1. Growth over time of (**A**) *Bacillus subtilis*, (**B**) *Bacillus thuringiensis* and (**C**) *Citrobacter rodentium* in LB medium (i.e. ' Control'), filter sterilized culture medium consiting out of 800 µl LB medium and 200 µl 10× diluted coelomic fluid ('Coelomic fluid') or culture medium containing both diluted coelomic fluid and antibiotics ('Antibiotics'). In the antibiotics treatments, Kanamycin (1µM) was used for *B. thuringiensis* and *B. subtilis* and Streptamycin (1 µM) was used for *C. rodentium*.

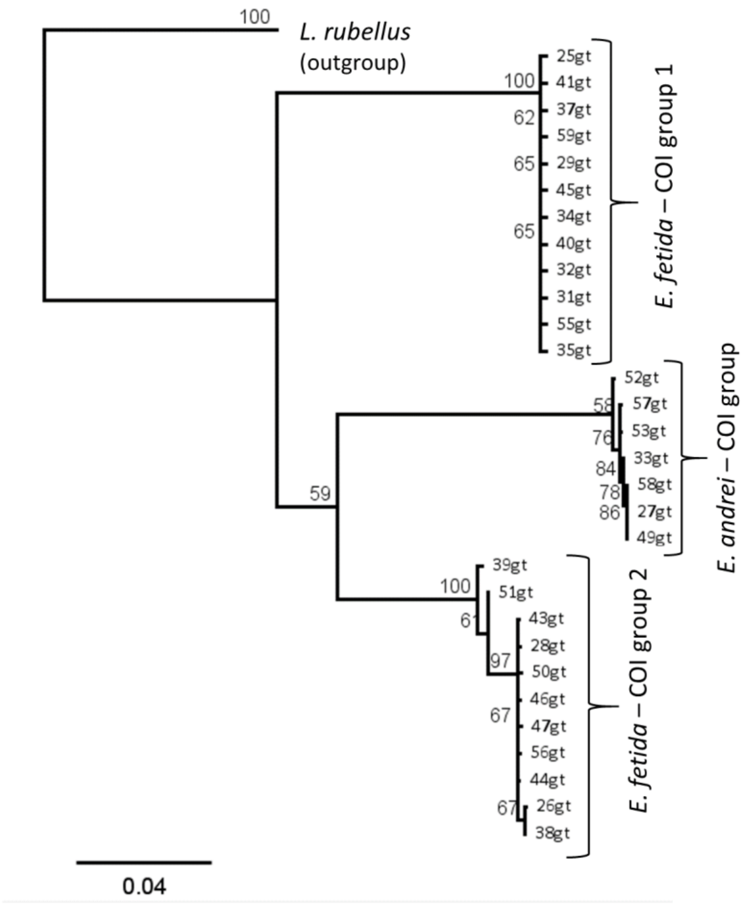

Figure S2. Maximum-likelihood phylogenetic tree of showing phylogenetic relation between all samples. Brackets indicate COI clusters. Bootstrap values are derived using 500 bootstraps.

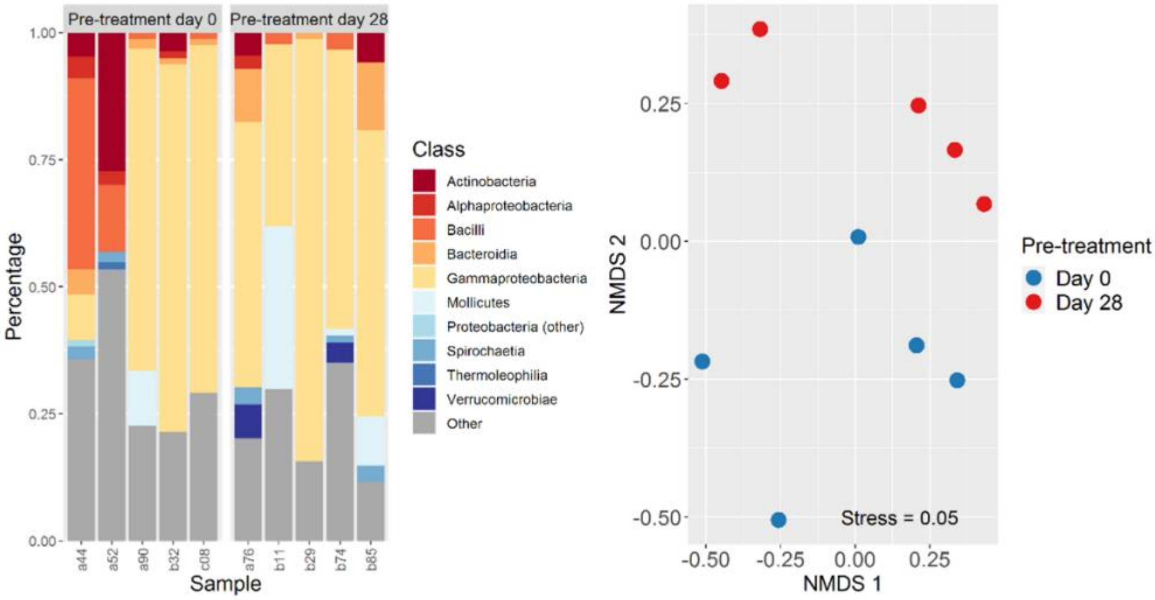

Figure S3. Bacterial community composition and structure of earthworm gut samples from control replicates at start and end of pre-treatment. (**A**) Relative abundance of dominant ASV at Class level per sample. All ASV with a relative abundance <1% of the total community are grouped under "Other". (**B**) Plot of NMDS at ASV level.

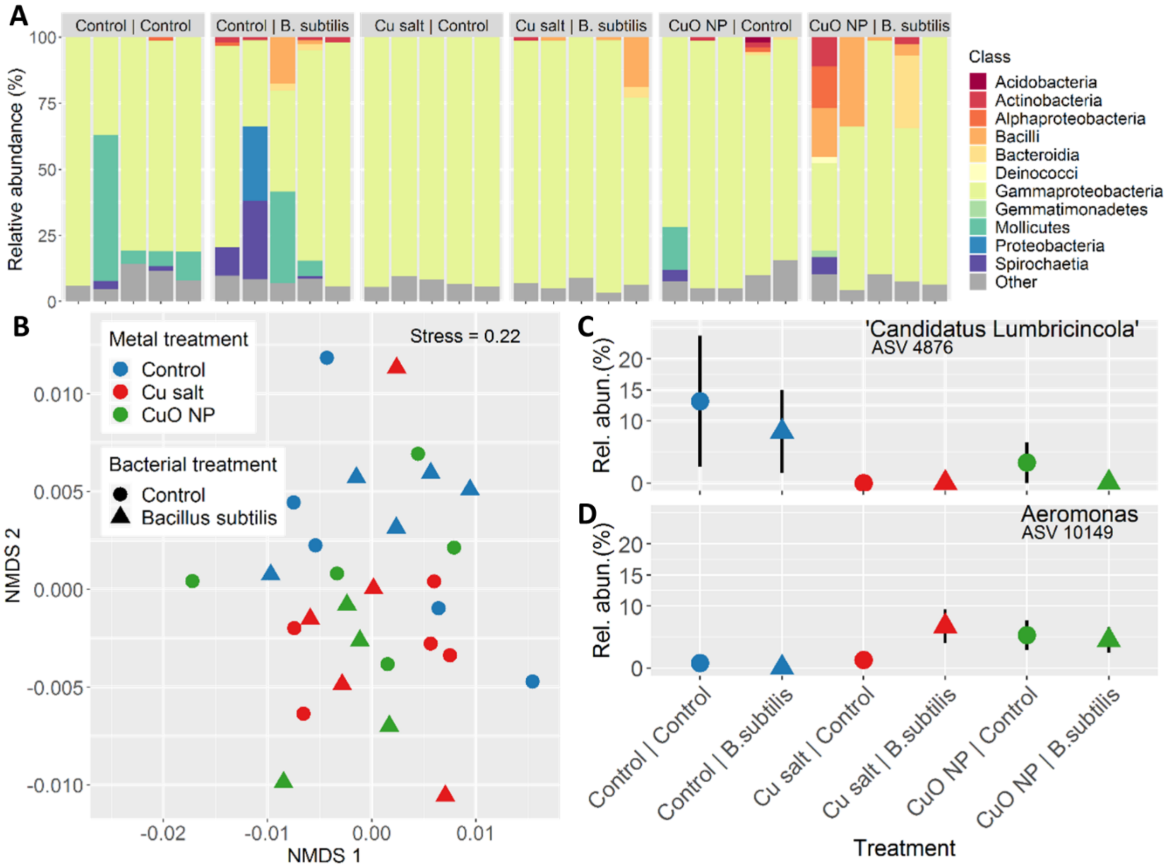

Figure S4. Bacterial community composition and structure of earthworm gut samples during bacterial challenge (i.e. 'Bacterial challenge day 2'). (**A**) Relative abundance of dominant ASV at Class level per sample. All ASV with a relative abundance <1% of the total community are grouped under "Other". (**B**) Plot of NMDS showing ordination of samples at ASV level. (**C–D**) Mean relative abundance (±se) per treatment as percentage of total community of '*Candidatus* Lumbricincola' (ASV 4876) and *Aeromonas* (ASV 10149).

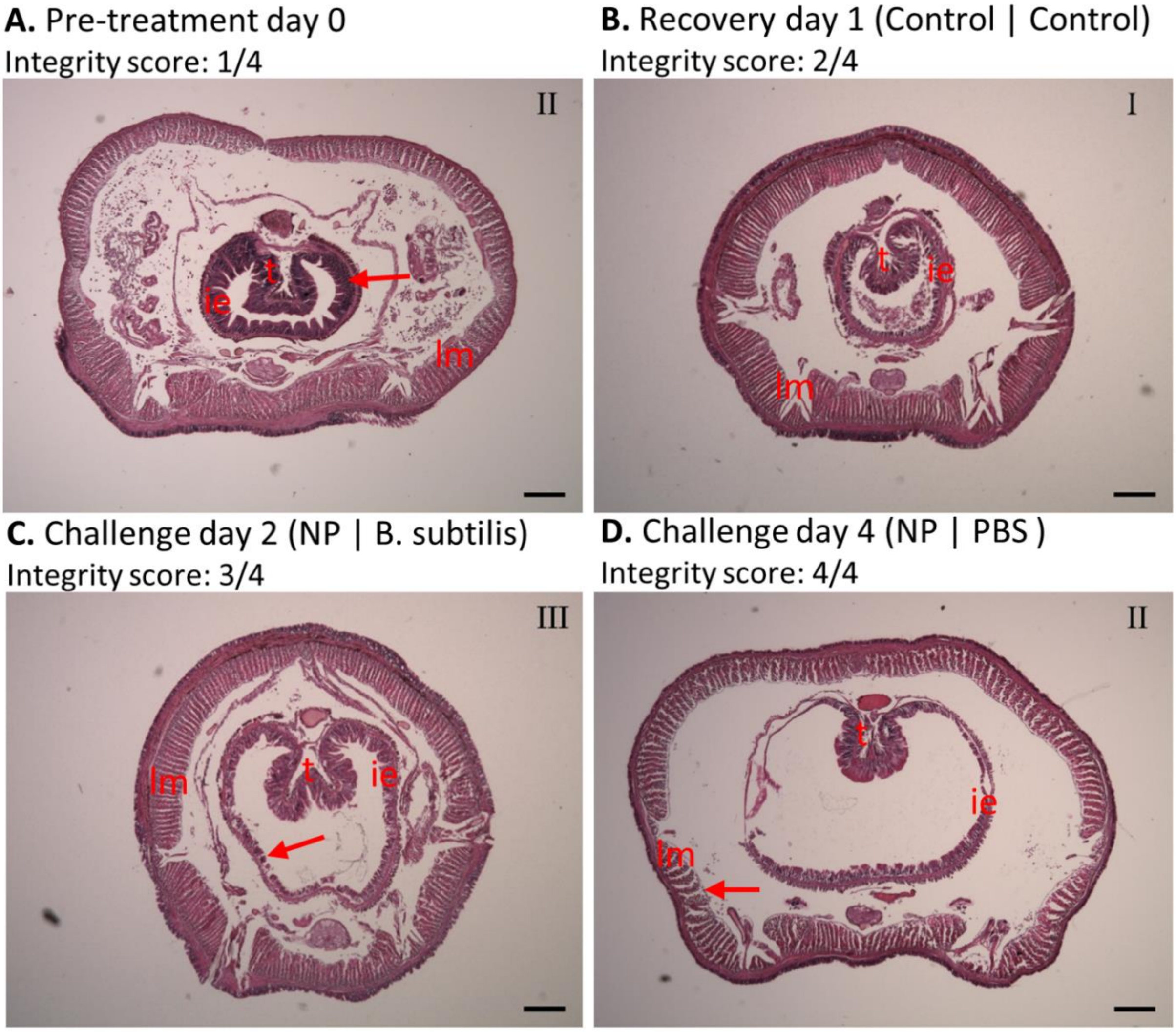

Figure S5. Examples of cross-sections of *E. fetida* stained with hematoxylin/eosin representing a range of tissue integrity scores. **A** represents a typical example of an undamaged specimen: a thick and dark colored intestinal lining (arrow). Damage to the intestine is manifested as the thinning the lining surrounding the intestinal epithelia as indicated by the arrow in **C**. A further indicator for tissue damage is the thinning of muscle fibers indicating necrosis of which a mild example is indicated by the arrow in **D.** Abbreviations: **t** typhlosole, **ie** intestinal epithelium and **lm** longitudinal muscles.

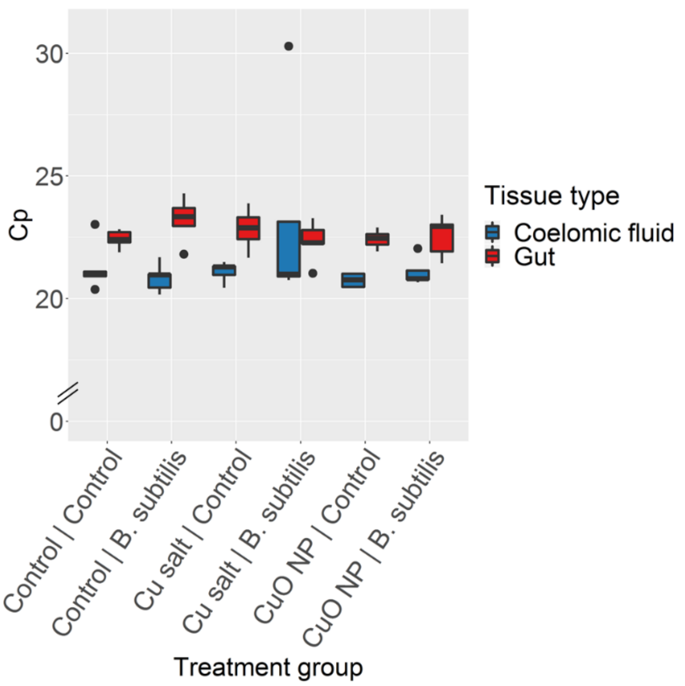

Figure S6. Boxplots of C_P values of elongation factor 1 alpha (ef1α) per treatment in coelomic fluid and gut tissue. On the x-axis, text before vertical bar indicates 'pre-treatment, text after vertical bar indicates 'bacterial challenge treatment'. No significant differences between treatment groups in C_P values were found for both coelomic fluid samples (F (5,54) = 1.071 p = 0.401) and gut tissue samples (F (5,24) = 1.37 p = 0.27). The outlier with C_P value ~30 in the 'Cu salt | B. subtilis' treatment group was removed from all further analysis.

Table S1. Mean Shannon diversity index and bacterial richness (±SD) per sampling point and treatment group.

**Diversity Index**	**Sampling Point**	**Bacterial Treatment: Control**			***B. subtilis***		
		**Pre-Treatment:**			**Pre-Treatment:**		
		Ctrl	NP	Ion	Ctrl	NP	Ion
Shannon Diversity	Pre-treatment (day 0)	3.3 ± 1.2					
	Pre-treatment (day 28)	2.6 ± 0.9	2.3 ± 1.1	2.6 ± 1.2			
	Bacterial challenge (day 2)	1.8 ± 0.8	2.3 ± 0.6	1.5 ± 0.6	1.9 ± 0.6	2.0 ± 0.4	2.3 ± 0.3
	Recovery period (day 1)	2.6 ± 0.3	3.3 ± 1.1	2.2 ± 0.7	2.2 ± 0.8	2.6 ± 1.1	2.7 ± 0.6
	Recovery period (day 28)	2.6 ± 1.2	2.1 ± 1.1	2.5 ± 1.1	2.5 ± 1.3	1.6 ± 0.7	2.6 ± 0.5
Bacterial Richness	Pre-treatment (day 0)	225 ± 167					
	Pre-treatment (day 28)	226 ± 226	115 ± 44	225 ± 118			
	Bacterial challenge (day 2)	62 ± 16	68 ± 41	49 ± 16	51 ± 10	53 ± 12	65 ± 31
	Recovery period (day 1)	118± 22	280 ± 192	134 ± 89	186 ± 137	184 ± 104	143 ± 96
	Recovery period (day 28)	210 ± 130	140 ± 105	154 ± 80	215 ± 231	78 ± 57	146 ± 19

Table S2. Integrity scores of gut epithelium and chloragogen tissue per sample point and treatment. Values are means of the scores of three sections from a single replicate.

Sampling Point	Bacterial Challenge Treatment: Control			Bacterial Challenge Treatment: *B. subtilis*		
	Pre-Treatment:			Pre-Treatment:		
	**Control**	**Cu salt**	**CuO NP**	**Control**	**Cu salt**	**CuO NP**
Pre-treatment (day 0)	1					
Pre-treatment (day 28)	1.3	1.3	1.0			
Bacterial challenge (day 2)	1.0	4.0	2.3	1.0	1.0	2.3
Bacterial challenge (day 4)	1.0	1.0	3.0	1.0	1.3	2.3
Recovery period (day 1)	2.0	2.0	1.7	2.0	3.3	3.3
Recovery period (day 28)	1.3	4.0	2.0	2.0	3.0	1.0
Average (across all sampling points)	1.3	2.5	2.0	1.5	2.2	2.2
